# Echocardiography in the ICU: an audit of 3 years practice

**DOI:** 10.1186/cc10799

**Published:** 2012-03-20

**Authors:** A Hall, J Walker, I Welters

**Affiliations:** 1 Royal Liverpool Hospital, Liverpool, UK

## Introduction

Assessment of the haemodynamically unstable patient is a core part of ICU management and relies predominantly on a combination of clinical skill and measurement of physiological variables. Echocardiography in the ICU has become increasingly popular as a tool for assessment of cardiac output, fluid status and ventricular function. Traditionally transoesophageal echo (TOE) has been favoured due to the belief that it gave superior images [1]. Transthoracic echo (TTE) is not often performed as it relies on 24-hour availability of trained personnel, availability of equipment and good patient windows [1]. There was also a perceived lack of benefit; however, recent studies have shown good or adequate images in over 85 to 90% of patients resulting in a change of management in 48% [1].

## Methods

Data were collected prospectively in all patients undergoing echocardiography in a teaching hospital ICU from January 2008 to January 2011. The main focus of our investigation was to ascertain the clinical questions to be answered and the outcome of echo on management.

## Results

A total of 238 echoes were performed on 216 patients with an average age of 59.75 years (TTE: 198, TOE: 19, and both: 14). The most commonly asked questions were on filling status and contractility (40%) and left ventricular function (33%). Ninety percent of clinical questions asked were answered fully (74%) or partially (16%) by echo. Sixty-one percent of echoes resulted in a change of management (5% of which were to continue with increased confidence). TTE performed by operators with basic training resulted in a 54% change in management. Changes included more filling (39%) and changes in inotropes or diuretics.

## Conclusion

Echocardiography in the ICU patient relies on numerous factors including skill and equipment availability and patient windows [1]. Our results confirm that there is a role for echo in these patients, important in a population where assessment of cardiac output and filling status is notoriously difficult. Our results also show that TTE performed by ICU physicians with basic training provides very useful information for the management of patients. This makes the focused courses on echocardiography very important [2,3]. Limitations of the study: an unknown amount of missing data and a likelihood of patient and operator bias as to which patients had echocardiography. In conclusion, echocardiography is a useful tool in the management of the haemodynamically unstable patients.
